# Poly[tris­(μ_3_-2-amino­ethane­sulfonato)­cobalt(II)potassium]

**DOI:** 10.1107/S1600536811039390

**Published:** 2011-09-30

**Authors:** Xiao-lin Li, Jing Yu, Yang-Miao Ou

**Affiliations:** a Physics and Chemistry Department, Jiangxi College of Traditional Chinese Medicine, Fuzhou, Jiangxi 344000, People’s Republic of China; b Department of Chemistry and Life Science, Hechi University, Yizhou, Guangxi 546300, People’s Republic of China

## Abstract

The title compound, [CoK(C_2_H_6_NO_3_S)_3_]_*n*_, is isotypic with its Ni^II^ analogue. The Co^II^ atom is chelated by the three taurinate ligands in a distorted octa­hedral geometry and in a facial manner. Each taurinate ligand bridges two K^+^ ions *via* its sulfonate group, forming a three-dimensional framework. Weak N—H⋯O hydrogen bonding is observed in the crystal structure.

## Related literature

For the isotypic Ni^II^ structure, see: Jiang *et al.* (2005 ▶). For the applications of taurine in medicine and biochemistry, see: Bottari & Festa (1998 ▶); Jiang *et al.* (2003 ▶). For general background to taurine complexes and their derivatives, see: Zhang & Jiang (2002 ▶); Zhong *et al.* (2003 ▶); Cai *et al.* (2004 ▶, 2006 ▶, 2011 ▶); Yang *et al.* (2010*a* ▶,*b* ▶). For S–O(–Co) bond lengths in bridging sulfonate groups, see: Zeng *et al.* (2009 ▶); Yang *et al.* (2010*b* ▶).
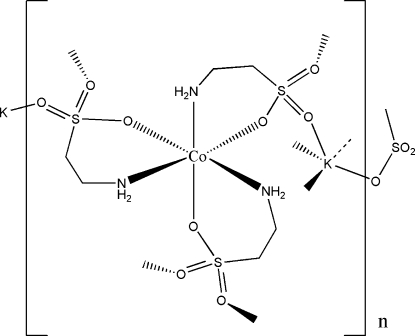


## Experimental

### 

#### Crystal data

[CoK(C_2_H_6_NO_3_S)_3_]
*M* *_r_* = 470.44
Orthorhombic, 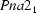

*a* = 10.6901 (19) Å
*b* = 15.669 (3) Å
*c* = 9.6094 (17) Å
*V* = 1609.6 (5) Å^3^
*Z* = 4
Mo *K*α radiation
μ = 1.76 mm^−1^
*T* = 296 K
0.26 × 0.22 × 0.14 mm

#### Data collection

Bruker SMART CCD area-detector diffractometer
Absorption correction: multi-scan (*SADABS*; Bruker, 1999 ▶) *T*_min_ = 0.657, *T*_max_ = 0.791
10792 measured reflections
3386 independent reflections
3146 reflections with *I* > 2σ(*I*)
*R*_int_ = 0.027

#### Refinement

*R*[*F* ^2^ > 2σ(*F* ^2^)] = 0.024
*wR*(*F* ^2^) = 0.058
*S* = 1.02
3386 reflections
208 parameters
1 restraint
H-atom parameters constrained
Δρ_max_ = 0.22 e Å^−3^
Δρ_min_ = −0.33 e Å^−3^
Absolute structure: Flack (1983 ▶), 1528 Friedel pairs
Flack parameter: 0.020 (13)

### 

Data collection: *SMART* (Bruker, 1999 ▶); cell refinement: *SAINT* (Bruker, 1999 ▶); data reduction: *SAINT*; program(s) used to solve structure: *SHELXS97* (Sheldrick, 2008 ▶); program(s) used to refine structure: *SHELXL97* (Sheldrick, 2008 ▶); molecular graphics: *SHELXTL* (Sheldrick, 2008 ▶); software used to prepare material for publication: *SHELXTL*.

## Supplementary Material

Crystal structure: contains datablock(s) I, global. DOI: 10.1107/S1600536811039390/bx2374sup1.cif

Structure factors: contains datablock(s) I. DOI: 10.1107/S1600536811039390/bx2374Isup2.hkl

Additional supplementary materials:  crystallographic information; 3D view; checkCIF report

## Figures and Tables

**Table 1:** Selected bond lengths (Å)

Co1—O1	2.1316 (18)
Co1—O7	2.1411 (18)
Co1—O4	2.142 (2)
Co1—N2	2.143 (2)
Co1—N3	2.146 (2)
Co1—N1	2.149 (2)
K1—O6^i^	2.687 (2)
K1—O3^ii^	2.7140 (19)
K1—O5^iii^	2.8361 (19)
K1—O2^iv^	2.8522 (19)
K1—O8^v^	2.8893 (19)
K1—O9	2.816 (2)

Symmetry codes: (i) 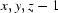; (ii) 
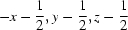
; (iii) 
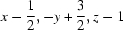
; (iv) 
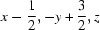
; (v) 
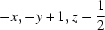
.

**Table 2:** Hydrogen-bond geometry (Å, °)

*D*—H⋯*A*	*D*—H	H⋯*A*	*D*⋯*A*	*D*—H⋯*A*
N3—H3*B*⋯O7^vi^	0.90	2.22	3.097 (3)	163
N2—H2*B*⋯O4^vi^	0.90	2.40	3.166 (3)	143
N2—H2*B*⋯O1^vi^	0.90	2.47	3.255 (3)	146
N1—H1*D*⋯O4^vi^	0.90	2.55	3.423 (3)	165
N3—H3*A*⋯O2	0.90	2.28	3.113 (3)	153
N2—H2*A*⋯O8	0.90	2.39	3.219 (3)	152
N1—H1*C*⋯O5	0.90	2.49	3.229 (3)	140

Symmetry code: (vi) 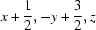.

**Fig. 1.**
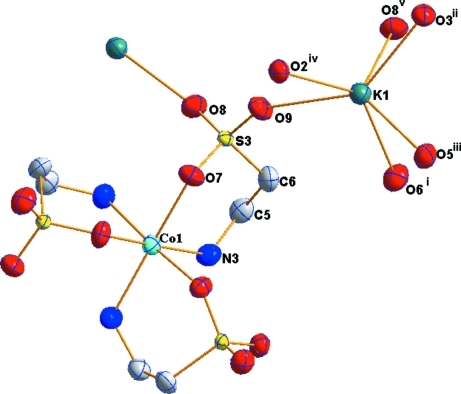
Molecular structure of the title compound.H atoms have been omitted for clarity. [Symmetry code: (i) x, y, z - 1; (ii) -x - 1/2, y - 1/2, z - 1/2; (iii) x - 1/2, -y + 3/2, z - 1; (iv) x - 1/2, -y + 3/2, z; (v) -x, -y + 1, z - 1/2.]

**Fig. 2.**
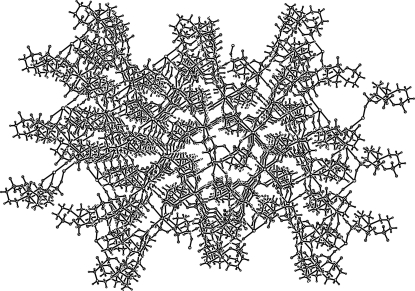
View of a three-dimensional supramolecular structure.

## supplementary crystallographic information

### Comment

Taurine, an amino acid containing sulfur, is indispensable to human beings Because of its applications in medicine and biochemistry (Bottari & Festa,1998; Jiang *et al.*,2003). Several taurine complexes and their derivatives have recently been prepared (Zhong *et al.* (2003); Cai *et al.* (2004, 2006, 2011); Yang *et al.* (2010*a*,*b*)). we found that the taurine has manifold coordination modes. For the much less well study of the coordination modes of the sulfonate group, The title polymeric Co^II^ complex,(I), has been prepared and its structure determined.

The coordinated modes of the title compound are similar to the previously reported Ni (II) structure (Jiang *et al.* (2005)). The molecular structure of (I) is shown in Fig. 1 and the important bond lengths are listed in Table 1. The asymmetric unit of (I) consists of one Co^II^ atom, three taurinate ligands and one K^+^ ion. The cobalt is six-coordinate with three nitrogen atoms [N(1), N(2), N(3)] and three oxygen atoms [O(1), O(4), O(7)], thus giving an octahedral configuration. The Co atom forms six membered chelate rings (NiNC_2_SO) with each taurinate ligand. This is a facial isomer. Each sulfonate group of the taurinate ligand takes part in the formation of a hydrogen bond (Table 2) with the amino group of a neighbouring ligand in the complex. The most common coordination modes of the sulfonate group are monodentate and µ_2_-bridging, while µ_3_-bridging is very rare. The coordination mode of the sulfonate group in (I) is µ_3_-bridging, which makes the S–O(–Co) bonds [1.475 (2)–1.479 (2) Å] much longer than those previously reported [S–O(–Co) 1.464 (2) and 1.456 (3) Å; Yang *et al.*, 2010*b*; Zeng *et al.*, 2009]. The S=O(···K) bonds [1.443 (2)–1.453 (2) Å] are slightly longer than the uncoordinated S=O bond in taurine [1.446 (12)–1.457 (13) Å; Zhang & Jiang, 2002]. The K atom is surrounded by six O atoms from different taurinate ligands, The title complex forms a three-dimensional structure through the K···O linkage. The K···O distances are in the range 2.678 (2)–2.889 (2) Å, suggesting weak electrostatic interactions.

### Experimental

A mixture of Co(CH_3_COO)_2_.7H_2_O (0.5 mmol, 152 mg), taurine (1.5 mmol 187 mg), KOH (1.5 mmol, 84 mg) and anhydrous methanol (15.0 ml) was placed in a Teflon-lined stainless steel vessel, and heated directly to 120 °C. After keeping at 120 °C for 4 days, it was cooled to room temperature at a rate for 10 °C/h. block red crystals of the complex were obtained.

### Refinement

H atoms were positioned geometrically (C–H = 0.97 Å and N–H = 0.90 Å) and included in the refinement in the riding model approximation, with *U*_iso_(H) = 1.2*U*_eq_(carrier atom).

### Crystal data

**Table:**

[CoK(C_2_H_6_NO_3_S)_3_]	*F*(000) = 964
*M**_r_* = 470.44	*D*_x_ = 1.941 Mg m^−^^3^
Orthorhombic, *P**n**a*2_1_	Mo *K*α radiation, λ = 0.71073 Å
Hall symbol: P 2c -2n	Cell parameters from 7233 reflections
*a* = 10.6901 (19) Å	θ = 2.5–28.1°
*b* = 15.669 (3) Å	µ = 1.76 mm^−^^1^
*c* = 9.6094 (17) Å	*T* = 296 K
*V* = 1609.6 (5) Å^3^	Block, red
*Z* = 4	0.26 × 0.22 × 0.14 mm

### Data collection

**Table:**

Bruker SMART CCD area-detector diffractometer	3386 independent reflections
Radiation source: fine-focus sealed tube	3146 reflections with *I* > 2σ(*I*)
graphite	*R*_int_ = 0.027
φ and ω scans	θ_max_ = 27.0°, θ_min_ = 2.3°
Absorption correction: multi-scan (*SADABS*; Bruker, 1999)	*h* = −13→13
*T*_min_ = 0.657, *T*_max_ = 0.791	*k* = −20→20
10792 measured reflections	*l* = −11→12

### Refinement

**Table:**

Refinement on *F*^2^	Secondary atom site location: difference Fourier map
Least-squares matrix: full	Hydrogen site location: inferred from neighbouring sites
*R*[*F*^2^ > 2σ(*F*^2^)] = 0.024	H-atom parameters constrained
*wR*(*F*^2^) = 0.058	*w* = 1/[σ^2^(*F*_o_^2^) + (0.0303*P*)^2^] where *P* = (*F*_o_^2^ + 2*F*_c_^2^)/3
*S* = 1.02	(Δ/σ)_max_ = 0.001
3386 reflections	Δρ_max_ = 0.22 e Å^−^^3^
208 parameters	Δρ_min_ = −0.33 e Å^−^^3^
1 restraint	Absolute structure: Flack (1983), 1528 Friedel pairs
Primary atom site location: structure-invariant direct methods	Flack parameter: 0.020 (13)

### Special details

**Table:**

Geometry. All e.s.d.'s (except the e.s.d. in the dihedral angle between two l.s. planes) are estimated using the full covariance matrix. The cell e.s.d.'s are taken into account individually in the estimation of e.s.d.'s in distances, angles and torsion angles; correlations between e.s.d.'s in cell parameters are only used when they are defined by crystal symmetry. An approximate (isotropic) treatment of cell e.s.d.'s is used for estimating e.s.d.'s involving l.s. planes.
Refinement. Refinement of *F*^2^ against ALL reflections. The weighted *R*-factor *wR* and goodness of fit *S* are based on *F*^2^, conventional *R*-factors *R* are based on *F*, with *F* set to zero for negative *F*^2^. The threshold expression of *F*^2^ > σ(*F*^2^) is used only for calculating *R*-factors(gt) *etc*. and is not relevant to the choice of reflections for refinement. *R*-factors based on *F*^2^ are statistically about twice as large as those based on *F*, and *R*- factors based on ALL data will be even larger.

### Fractional atomic coordinates and isotropic or equivalent isotropic displacement parameters (Å^2^)

**Table:**

	*x*	*y*	*z*	*U*_iso_*/*U*_eq_	
Co1	0.11671 (3)	0.758726 (19)	0.15941 (5)	0.02649 (9)	
S1	0.02451 (5)	0.93821 (4)	0.02671 (7)	0.02606 (14)	
S2	0.01351 (6)	0.72499 (4)	0.46938 (7)	0.02860 (14)	
S3	0.02648 (6)	0.60549 (4)	−0.04504 (7)	0.02819 (14)	
O1	−0.00760 (15)	0.85153 (11)	0.0747 (2)	0.0323 (4)	
O4	−0.01485 (17)	0.75606 (12)	0.3275 (2)	0.0354 (5)	
C1	0.1093 (2)	0.98601 (17)	0.1650 (4)	0.0370 (6)	
H1A	0.1248	1.0453	0.1416	0.044*	
H1B	0.0575	0.9852	0.2479	0.044*	
O7	−0.00597 (15)	0.66745 (11)	0.0653 (2)	0.0341 (4)	
N3	0.2342 (2)	0.76329 (15)	−0.0215 (3)	0.0352 (5)	
H3A	0.2121	0.8104	−0.0691	0.042*	
H3B	0.3127	0.7721	0.0091	0.042*	
O9	−0.08429 (17)	0.57634 (13)	−0.1176 (2)	0.0405 (5)	
O6	−0.10014 (17)	0.70601 (14)	0.5464 (2)	0.0435 (5)	
O5	0.09764 (18)	0.78116 (13)	0.5428 (2)	0.0410 (5)	
N2	0.2285 (2)	0.65745 (15)	0.2430 (3)	0.0353 (5)	
H2A	0.2123	0.6108	0.1915	0.042*	
H2B	0.3089	0.6714	0.2269	0.042*	
N1	0.2190 (2)	0.85854 (15)	0.2622 (3)	0.0406 (6)	
H1C	0.1828	0.8664	0.3458	0.049*	
H1D	0.2964	0.8383	0.2785	0.049*	
O2	0.10597 (16)	0.93593 (13)	−0.0947 (2)	0.0335 (4)	
O3	−0.08694 (16)	0.98967 (12)	0.0079 (2)	0.0372 (5)	
O8	0.10532 (17)	0.53775 (12)	0.0077 (2)	0.0400 (5)	
C2	0.2341 (2)	0.94364 (17)	0.1992 (3)	0.0358 (7)	
H2C	0.2828	0.9383	0.1144	0.043*	
H2D	0.2803	0.9799	0.2628	0.043*	
C4	0.2203 (3)	0.6309 (2)	0.3918 (3)	0.0405 (7)	
H4A	0.2601	0.5756	0.4027	0.049*	
H4B	0.2658	0.6716	0.4486	0.049*	
C5	0.2410 (2)	0.69305 (19)	−0.1240 (3)	0.0388 (7)	
H5A	0.2858	0.6455	−0.0830	0.047*	
H5B	0.2882	0.7122	−0.2044	0.047*	
C6	0.1144 (3)	0.6630 (2)	−0.1711 (3)	0.0400 (7)	
H6A	0.1253	0.6269	−0.2522	0.048*	
H6B	0.0660	0.7123	−0.1998	0.048*	
C3	0.0887 (3)	0.62542 (17)	0.4424 (3)	0.0391 (7)	
H3C	0.0881	0.5940	0.5294	0.047*	
H3D	0.0403	0.5929	0.3755	0.047*	
K1	−0.25179 (5)	0.58729 (4)	−0.34300 (7)	0.03377 (13)	

### Atomic displacement parameters (Å^2^)

**Table:**

	*U*^11^	*U*^22^	*U*^33^	*U*^12^	*U*^13^	*U*^23^
Co1	0.02482 (16)	0.02763 (16)	0.02701 (17)	−0.00158 (13)	−0.00214 (15)	0.00258 (18)
S1	0.0264 (3)	0.0271 (3)	0.0247 (3)	0.0020 (2)	0.0012 (3)	0.0002 (3)
S2	0.0305 (3)	0.0299 (3)	0.0255 (3)	−0.0029 (3)	−0.0005 (3)	0.0035 (3)
S3	0.0263 (3)	0.0273 (3)	0.0310 (3)	−0.0002 (2)	−0.0040 (3)	−0.0012 (3)
O1	0.0275 (9)	0.0322 (9)	0.0373 (11)	−0.0025 (8)	−0.0026 (8)	0.0060 (9)
O4	0.0347 (10)	0.0446 (12)	0.0269 (10)	0.0049 (9)	−0.0010 (8)	0.0091 (9)
C1	0.0433 (14)	0.0354 (13)	0.0324 (15)	0.0022 (12)	−0.0045 (13)	−0.0100 (16)
O7	0.0277 (9)	0.0327 (10)	0.0419 (12)	−0.0020 (8)	0.0015 (8)	−0.0095 (9)
N3	0.0294 (11)	0.0359 (12)	0.0403 (14)	−0.0045 (9)	0.0053 (10)	0.0031 (11)
O9	0.0356 (11)	0.0411 (11)	0.0448 (13)	−0.0029 (9)	−0.0125 (9)	−0.0060 (10)
O6	0.0357 (10)	0.0525 (13)	0.0424 (12)	−0.0065 (9)	0.0102 (9)	0.0090 (11)
O5	0.0464 (11)	0.0388 (11)	0.0378 (12)	−0.0084 (9)	−0.0056 (9)	−0.0048 (10)
N2	0.0315 (12)	0.0382 (13)	0.0362 (15)	0.0055 (10)	−0.0006 (10)	0.0017 (11)
N1	0.0420 (13)	0.0383 (13)	0.0416 (14)	−0.0097 (11)	−0.0125 (11)	0.0057 (12)
O2	0.0372 (10)	0.0369 (11)	0.0262 (10)	0.0008 (8)	0.0059 (8)	0.0018 (8)
O3	0.0343 (10)	0.0384 (11)	0.0388 (12)	0.0092 (8)	0.0005 (8)	0.0011 (9)
O8	0.0388 (11)	0.0319 (10)	0.0492 (14)	0.0076 (8)	−0.0064 (9)	0.0028 (10)
C2	0.0349 (15)	0.0383 (15)	0.0343 (16)	−0.0077 (12)	−0.0045 (11)	−0.0006 (12)
C4	0.0419 (16)	0.0382 (16)	0.0414 (18)	0.0080 (13)	−0.0072 (13)	0.0039 (13)
C5	0.0349 (15)	0.0422 (17)	0.0395 (17)	0.0007 (13)	0.0072 (12)	0.0031 (14)
C6	0.0484 (17)	0.0439 (17)	0.0277 (16)	−0.0019 (14)	−0.0015 (12)	−0.0001 (13)
C3	0.0534 (18)	0.0281 (14)	0.0357 (17)	0.0001 (12)	−0.0019 (14)	0.0061 (13)
K1	0.0351 (3)	0.0354 (3)	0.0309 (3)	−0.0048 (2)	−0.0014 (3)	0.0009 (3)

### Geometric parameters (Å, °)

**Table:**

Co1—O1	2.1316 (18)	N2—C4	1.492 (4)
Co1—O7	2.1411 (18)	N2—H2A	0.9000
Co1—O4	2.142 (2)	N2—H2B	0.9000
Co1—N2	2.143 (2)	N1—C2	1.473 (4)
Co1—N3	2.146 (2)	N1—H1C	0.9000
Co1—N1	2.149 (2)	N1—H1D	0.9000
S1—O3	1.4500 (18)	O2—K1^iii^	2.8522 (19)
S1—O2	1.4567 (19)	O3—K1^iv^	2.7140 (19)
S1—O1	1.4748 (18)	O8—K1^v^	2.8893 (19)
S1—C1	1.775 (3)	C2—H2C	0.9700
S2—O5	1.443 (2)	C2—H2D	0.9700
S2—O6	1.4533 (19)	C4—C3	1.491 (4)
S2—O4	1.479 (2)	C4—H4A	0.9700
S2—C3	1.774 (3)	C4—H4B	0.9700
S3—O8	1.4472 (19)	C5—C6	1.503 (4)
S3—O9	1.4482 (19)	C5—H5A	0.9700
S3—O7	1.479 (2)	C5—H5B	0.9700
S3—C6	1.778 (3)	C6—H6A	0.9700
C1—C2	1.525 (4)	C6—H6B	0.9700
C1—H1A	0.9700	C3—H3C	0.9700
C1—H1B	0.9700	C3—H3D	0.9700
N3—C5	1.479 (4)	K1—O6^vi^	2.687 (2)
N3—H3A	0.9000	K1—O3^vii^	2.7140 (19)
N3—H3B	0.9000	K1—O5^viii^	2.8361 (19)
O9—K1	2.816 (2)	K1—O2^ix^	2.8522 (19)
O6—K1^i^	2.687 (2)	K1—O8^x^	2.8893 (19)
O5—K1^ii^	2.8361 (19)	K1—O9	2.816 (2)
			
O1—Co1—O7	84.97 (7)	H2A—N2—H2B	106.6
O1—Co1—O4	83.80 (7)	C2—N1—Co1	121.71 (18)
O7—Co1—O4	84.46 (7)	C2—N1—H1C	106.9
O1—Co1—N2	174.95 (8)	Co1—N1—H1C	106.9
O7—Co1—N2	90.31 (8)	C2—N1—H1D	106.9
O4—Co1—N2	93.97 (8)	Co1—N1—H1D	106.9
O1—Co1—N3	91.86 (9)	H1C—N1—H1D	106.7
O7—Co1—N3	92.19 (8)	S1—O2—K1^iii^	172.74 (12)
O4—Co1—N3	174.73 (8)	S1—O3—K1^iv^	140.30 (12)
N2—Co1—N3	90.11 (9)	S3—O8—K1^v^	170.75 (13)
O1—Co1—N1	89.78 (8)	N1—C2—C1	112.7 (2)
O7—Co1—N1	172.81 (8)	N1—C2—H2C	109.0
O4—Co1—N1	90.10 (9)	C1—C2—H2C	109.0
N2—Co1—N1	94.76 (10)	N1—C2—H2D	109.0
N3—Co1—N1	92.89 (9)	C1—C2—H2D	109.0
O3—S1—O2	113.88 (12)	H2C—C2—H2D	107.8
O3—S1—O1	111.10 (11)	C3—C4—N2	112.6 (2)
O2—S1—O1	111.52 (12)	C3—C4—H4A	109.1
O3—S1—C1	106.18 (12)	N2—C4—H4A	109.1
O2—S1—C1	107.75 (13)	C3—C4—H4B	109.1
O1—S1—C1	105.88 (13)	N2—C4—H4B	109.1
O5—S2—O6	113.38 (13)	H4A—C4—H4B	107.8
O5—S2—O4	112.20 (13)	N3—C5—C6	112.9 (2)
O6—S2—O4	111.44 (12)	N3—C5—H5A	109.0
O5—S2—C3	108.98 (13)	C6—C5—H5A	109.0
O6—S2—C3	105.87 (13)	N3—C5—H5B	109.0
O4—S2—C3	104.34 (14)	C6—C5—H5B	109.0
O8—S3—O9	114.43 (12)	H5A—C5—H5B	107.8
O8—S3—O7	111.54 (13)	C5—C6—S3	115.5 (2)
O9—S3—O7	111.12 (12)	C5—C6—H6A	108.4
O8—S3—C6	107.61 (13)	S3—C6—H6A	108.4
O9—S3—C6	105.31 (14)	C5—C6—H6B	108.4
O7—S3—C6	106.24 (13)	S3—C6—H6B	108.4
S1—O1—Co1	127.05 (10)	H6A—C6—H6B	107.5
S2—O4—Co1	124.53 (11)	C4—C3—S2	115.1 (2)
C2—C1—S1	115.1 (2)	C4—C3—H3C	108.5
C2—C1—H1A	108.5	S2—C3—H3C	108.5
S1—C1—H1A	108.5	C4—C3—H3D	108.5
C2—C1—H1B	108.5	S2—C3—H3D	108.5
S1—C1—H1B	108.5	H3C—C3—H3D	107.5
H1A—C1—H1B	107.5	O6^vi^—K1—O3^vii^	124.38 (7)
S3—O7—Co1	126.71 (10)	O6^vi^—K1—O9	87.87 (6)
C5—N3—Co1	122.94 (17)	O3^vii^—K1—O9	140.88 (6)
C5—N3—H3A	106.6	O6^vi^—K1—O5^viii^	71.71 (6)
Co1—N3—H3A	106.6	O3^vii^—K1—O5^viii^	81.07 (6)
C5—N3—H3B	106.6	O9—K1—O5^viii^	134.66 (7)
Co1—N3—H3B	106.6	O6^vi^—K1—O2^ix^	137.80 (7)
H3A—N3—H3B	106.6	O3^vii^—K1—O2^ix^	91.78 (6)
S3—O9—K1	150.60 (13)	O9—K1—O2^ix^	71.81 (6)
S2—O6—K1^i^	147.95 (13)	O5^viii^—K1—O2^ix^	96.53 (6)
S2—O5—K1^ii^	170.53 (14)	O6^vi^—K1—O8^x^	86.91 (7)
C4—N2—Co1	122.22 (18)	O3^vii^—K1—O8^x^	72.54 (6)
C4—N2—H2A	106.8	O9—K1—O8^x^	89.77 (6)
Co1—N2—H2A	106.8	O5^viii^—K1—O8^x^	127.47 (7)
C4—N2—H2B	106.8	O2^ix^—K1—O8^x^	128.14 (6)
Co1—N2—H2B	106.8		
			
O3—S1—O1—Co1	−165.99 (13)	O4—S2—O5—K1^ii^	24.0 (8)
O2—S1—O1—Co1	65.79 (17)	C3—S2—O5—K1^ii^	139.1 (8)
C1—S1—O1—Co1	−51.14 (17)	O1—Co1—N2—C4	−81.5 (11)
O7—Co1—O1—S1	−148.59 (16)	O7—Co1—N2—C4	−102.3 (2)
O4—Co1—O1—S1	126.45 (16)	O4—Co1—N2—C4	−17.8 (2)
N2—Co1—O1—S1	−169.5 (9)	N3—Co1—N2—C4	165.5 (2)
N3—Co1—O1—S1	−56.56 (16)	N1—Co1—N2—C4	72.6 (2)
N1—Co1—O1—S1	36.33 (17)	O1—Co1—N1—C2	−34.1 (2)
O5—S2—O4—Co1	69.55 (17)	O7—Co1—N1—C2	−77.2 (8)
O6—S2—O4—Co1	−162.09 (13)	O4—Co1—N1—C2	−117.9 (2)
C3—S2—O4—Co1	−48.29 (17)	N2—Co1—N1—C2	148.1 (2)
O1—Co1—O4—S2	−159.21 (15)	N3—Co1—N1—C2	57.7 (2)
O7—Co1—O4—S2	115.26 (15)	O3—S1—O2—K1^iii^	−91.5 (9)
N2—Co1—O4—S2	25.34 (16)	O1—S1—O2—K1^iii^	35.3 (9)
N3—Co1—O4—S2	166.0 (8)	C1—S1—O2—K1^iii^	151.0 (9)
N1—Co1—O4—S2	−69.44 (15)	O2—S1—O3—K1^iv^	−149.88 (15)
O3—S1—C1—C2	−177.7 (2)	O1—S1—O3—K1^iv^	83.18 (19)
O2—S1—C1—C2	−55.3 (3)	C1—S1—O3—K1^iv^	−31.5 (2)
O1—S1—C1—C2	64.1 (2)	O9—S3—O8—K1^v^	−114.2 (7)
O8—S3—O7—Co1	71.69 (17)	O7—S3—O8—K1^v^	13.0 (8)
O9—S3—O7—Co1	−159.33 (13)	C6—S3—O8—K1^v^	129.1 (7)
C6—S3—O7—Co1	−45.30 (17)	Co1—N1—C2—C1	56.8 (3)
O1—Co1—O7—S3	117.83 (15)	S1—C1—C2—N1	−69.4 (3)
O4—Co1—O7—S3	−157.93 (15)	Co1—N2—C4—C3	43.5 (3)
N2—Co1—O7—S3	−63.97 (16)	Co1—N3—C5—C6	49.2 (3)
N3—Co1—O7—S3	26.15 (16)	N3—C5—C6—S3	−71.0 (3)
N1—Co1—O7—S3	161.1 (7)	O8—S3—C6—C5	−52.0 (3)
O1—Co1—N3—C5	−108.5 (2)	O9—S3—C6—C5	−174.4 (2)
O7—Co1—N3—C5	−23.5 (2)	O7—S3—C6—C5	67.6 (2)
O4—Co1—N3—C5	−73.9 (9)	N2—C4—C3—S2	−73.2 (3)
N2—Co1—N3—C5	66.8 (2)	O5—S2—C3—C4	−45.3 (3)
N1—Co1—N3—C5	161.6 (2)	O6—S2—C3—C4	−167.6 (2)
O8—S3—O9—K1	−136.5 (2)	O4—S2—C3—C4	74.7 (2)
O7—S3—O9—K1	96.0 (3)	S3—O9—K1—O6^vi^	4.6 (3)
C6—S3—O9—K1	−18.6 (3)	S3—O9—K1—O3^vii^	152.8 (2)
O5—S2—O6—K1^i^	−123.5 (2)	S3—O9—K1—O5^viii^	−56.8 (3)
O4—S2—O6—K1^i^	108.8 (2)	S3—O9—K1—O2^ix^	−137.8 (3)
C3—S2—O6—K1^i^	−4.1 (3)	S3—O9—K1—O8^x^	91.6 (3)
O6—S2—O5—K1^ii^	−103.3 (8)		

Symmetry codes: (i) *x*, *y*, *z*+1; (ii) *x*+1/2, −*y*+3/2, *z*+1; (iii) *x*+1/2, −*y*+3/2, *z*; (iv) −*x*−1/2, *y*+1/2, *z*+1/2; (v) −*x*, −*y*+1, *z*+1/2; (vi) *x*, *y*, *z*−1; (vii) −*x*−1/2, *y*−1/2, *z*−1/2; (viii) *x*−1/2, −*y*+3/2, *z*−1; (ix) *x*−1/2, −*y*+3/2, *z*; (x) −*x*, −*y*+1, *z*−1/2.

### Hydrogen-bond geometry (Å, °)

**Table:**

*D*—H···*A*	*D*—H	H···*A*	*D*···*A*	*D*—H···*A*
N3—H3B···O7^iii^	0.90	2.22	3.097 (3)	163.
N2—H2B···O4^iii^	0.90	2.40	3.166 (3)	143.
N2—H2B···O1^iii^	0.90	2.47	3.255 (3)	146.
N1—H1D···O4^iii^	0.90	2.55	3.423 (3)	165.
N3—H3A···O2	0.90	2.28	3.113 (3)	153.
N2—H2A···O8	0.90	2.39	3.219 (3)	152.
N1—H1C···O5	0.90	2.49	3.229 (3)	140.

Symmetry codes: (iii) *x*+1/2, −*y*+3/2, *z*.
